# Transcriptome analysis unveils survival strategies of *Streptococcus parauberis* against fish serum

**DOI:** 10.1371/journal.pone.0252200

**Published:** 2021-05-26

**Authors:** Yoonhang Lee, Nameun Kim, HyeongJin Roh, Ahran Kim, Hyun-Ja Han, Miyoung Cho, Do-Hyung Kim

**Affiliations:** 1 Department of Aquatic Life Medicine, Pukyong National University, Busan, Republic of Korea; 2 Pathology Research Division, National Institute of Fisheries Science, Busan, Republic of Korea; University of Graz, AUSTRIA

## Abstract

*Streptococcus parauberis* is an important bacterial fish pathogen that causes streptococcosis in a variety of fish species including the olive flounder. Despite its importance in the aquaculture industry, little is known about the survival strategy of *S*. *parauberis* in the host. Therefore, the objective of this study was to produce genome-wide transcriptome data and identify key factors for the survival of *S*. *parauberis* SPOF3K in its host. To this end, *S*. *parauberis* SPOF3K was incubated in olive flounder serum and nutrient-enriched media as a control. Although *S*. *parauberis* SPOF3K proliferated in both culture conditions, the transcriptomic patterns of the two groups were very different. Interestingly, the expression levels of genes responsible for the replication of an *S*. *parauberis* plasmid in the presence of olive flounder serum were higher than those in the absence of olive flounder serum, indicating that this plasmid may play an important role in the survival and proliferation of *S*. *parauberis* in the host. Several ATP-binding cassette transporters known to transport organic substrates (e.g., biotin and osmoprotectants) that are vital for bacterial survival in the host were significantly up-regulated in *S*. *parauberis* cultured in serum. In addition, *groEL*, *dnaK* operon, and members of the *clp* protease family, which are known to play important roles in response to various stressors, were up-regulated in *S*. *parauberis* incubated in serum, thus limiting damage and facilitating cellular recovery. Moreover, important virulence factors including the hyaluronic acid capsule (*has* operon), sortase A (s*rtA*), C5a peptidase (*scp*), and peptidoglycan O-acetyltransferase (*oatA*) were significantly upregulated in *S*. *paraubers* in serum. These results indicate that *S*. *paraubers* can resist and evade the humoral immune responses of fish. The transcriptomic data obtained in this study provide a better understanding of the mode of action of *S*. *parauberis* in fish.

## Introduction

Streptococcosis is one of the most important bacterial diseases in a number of fish species worldwide [1]. In South Korea, streptococcosis caused by *Streptococcus parauberis* is the dominant bacterial disease in the olive flounder (*Paralichthys olivacus*) [2]. In an effort to reduce the devastating impact of the disease on the aquaculture industry, studies have addressed diagnostic sensitivity [3], antibiotic efficacy [4], serotype variations [5], and development of an inactivated whole-cell vaccine [6]. Also, it has been revealed that the heart and brain are the major pathological target organs of *S*. *parauberis*; thus, the microbe can cause pericarditis and/or meningitis, leading to mortality, in the olive flounder [7]. Despite these academic findings and the many practical applications of research in the field, Streptococcosis remains the most common and threatening bacterial disease in South Korean aquaculture farms [2]. Recently, several genomic studies have been done on *S*. *parauberis* derived from a variety of fish species including the olive flounder and the data is being made publicly available [8, 9]. However, there is a limited amount of experimental data on functional characterization of genetic components. In addition, no efforts have yet been made to identify the pathogenic mechanism or key virulence factors of *S*. *parauberis*.

In recent years, transcriptomic analysis using massively parallel cDNA sequencing (RNA-seq) techniques has commonly been used to understand the pathogenesis of various types of pathogens. *Ex vivo* models are used for this purpose, which study bacterial cultures in various environments such as milk [10], saliva [11], heparinized blood [12], and host serum [12–16]. Of these, serum may be the best option for furthering our understanding of microbial pathogenesis as it contains various humoral immune factors including complements, lysozymes, antitoxins, bacteriolysins, bacterial agglutinins, and antimicrobial peptides [17]. Previous transcriptomic data from bacterial cultures in host serum have revealed key factors involved in bacterial survival and adaptation to the humoral immune system [12–16]. For example, Huja et al. [14] showed that Fur is a major regulatory protein involved in pathogenic *Escherichia coli* resistance against human serum by screening for transcriptomic and proteomic alterations after exposure of the bacteria to the serum.

In this study, therefore, global transcriptome analysis was performed to investigate how *S*. *parauberis* survives in the presence of host immunity by systemically monitoring gene expression alterations after exposure to olive flounder serum, and to identify key factors mediating its survival and adaptation strategy in the host. The novel findings of this study will aid in better understanding the pathogenicity of this organism and developing prophylactic strategies.

## Material and methods

### Experimental design and culture conditions

The SPOF3K, virulent strain of *Streptococcus parauberis* used in this study was isolated from the kidney of a diseased olive flounder (*Paralichthys olivaceus*) in Geojedo, Korea in 2013. The genome of strain SPOF3K was sequenced, annotated, and demonstrated in our previous study [9]. Healthy one-year-old olive flounders weighing approximately 200 g were purchased from a commercial fish farm in Busan, South Korea. Olive flounder blood was drawn from three individual fish that had been anesthetized with Ethyl 3-aminobenzoate methanesulfonate (MS-222; Sigma-Aldrich, St Louis, MO, USA). This study was approved by Ethics Committee of Pukyong National University (approval number: 2017–10) according to the Bioethics and Safety Act of the South Korean Ministry of Health and Welfare. Serum was separated from the blood by centrifugation at 6500 rpm at 4°C. Bacteria were cultured in brain heart infusion broth (Becton Dickinson, Franklin Lakes, New Jersey, USA) supplemented with 1% NaCl (BN) at 26°C for 18 hours with shaking at 160 rpm. Bacterial cells were harvested at the early-stationary phase and washed with phosphate-buffered saline (PBS, pH 7.4 at 25°C) by centrifugation at 6000 rpm for 10 min at room temperature. The bacterial culture was then resuspended in PBS, mixed with an equal volume of BN medium or olive flounder serum (final concentration, 10^9^ CFU ml^-1^) and incubated at 26°C for four hours with gentle shaking. All samples were prepared in triplicate. At 0, 1, 2 and 4 hours post-incubation, bacterial cells were sampled for RNA extraction and viable bacterial cells were counted by the plate counting method. Student’s t-test was used to identify significant differences (*p*-value < 0.05) in viable cell counts under different culture conditions.

### RNA-sequencing analysis

Total RNA was extracted from bacterial pellets using the RiboPureTM Bacteria kit (Ambion Life Technologies, Grand Island, NY, USA) according to the manufacturer’s instructions. Samples were treated with DNase I (Ambion Life Technologies) to remove trace amounts of genomic DNA. RNA concentration and quality were determined using a Qubit 3 Fluorometer and RNA High-Sensitivity Assay kit (Invitrogen, Carlsbad, CA, USA) and an Agilent 2100 Bioanalyzer (Agilent Technologies, Santa Clara, CA), respectively. Ribosomal RNA in the samples was depleted using a Ribo-zero-rRNA Removal Kit (Epicentre, Madison, WI, USA). cDNA libraries were constructed using a TruSeq RNA Sample Prep Kit (Illumina, Sand Diego, CA, USA) (insertion size > 250 bp) and sequenced using an Illumina Hiseq 2500 (1 × 50 nucleotide read length). Raw Illumina sequence reads were evaluated and trimmed using FastQC [18] to remove low-quality reads. The remaining reads were mapped onto a reference SPOF3K genome [9] (accession numbers: CP025420.1, CP025421.1) using Bowtie2 [19]. DESeq2 [20] was used for data normalization and differential gene expression (DGE) analysis by uploading read counts for each gene into iDEP.92 [21]. The statistical significance of DGE was calculated based on fold change (significance at |fold change| > 1.5) and the false discovery rate (FDR, significance at < 1e^-5^) according to Benjamini and Hochberg [22]. Principle component analysis (PCA), hierarchical and K-means clustering analysis, Venn diagram analysis, and visualization were accomplished using iDEP.92 and Morpheus (https://software.broadistitute.org/morpheus). Transcripts of broth-cultured samples were used as controls for the serum-cultured samples at every sampling time point. To measure gene expression profiles based on eggNOG functional categories [23], *z*-score was calculated by dividing the number of significantly up- and down-regulated genes (with consideration of FDR only, significance at < 1e^-5^) by the square of the total number of differentially expressed genes (DEGs) [24].

$$z-score=\frac{\left(Up\;regulated\;DEG\;count)-(Down\;regulated\;DEG\;count\right)}{\sqrt{Total\;DEG\;count}}$$

## Results and discussion

### Bacterial survival in serum

**Fig 1:**
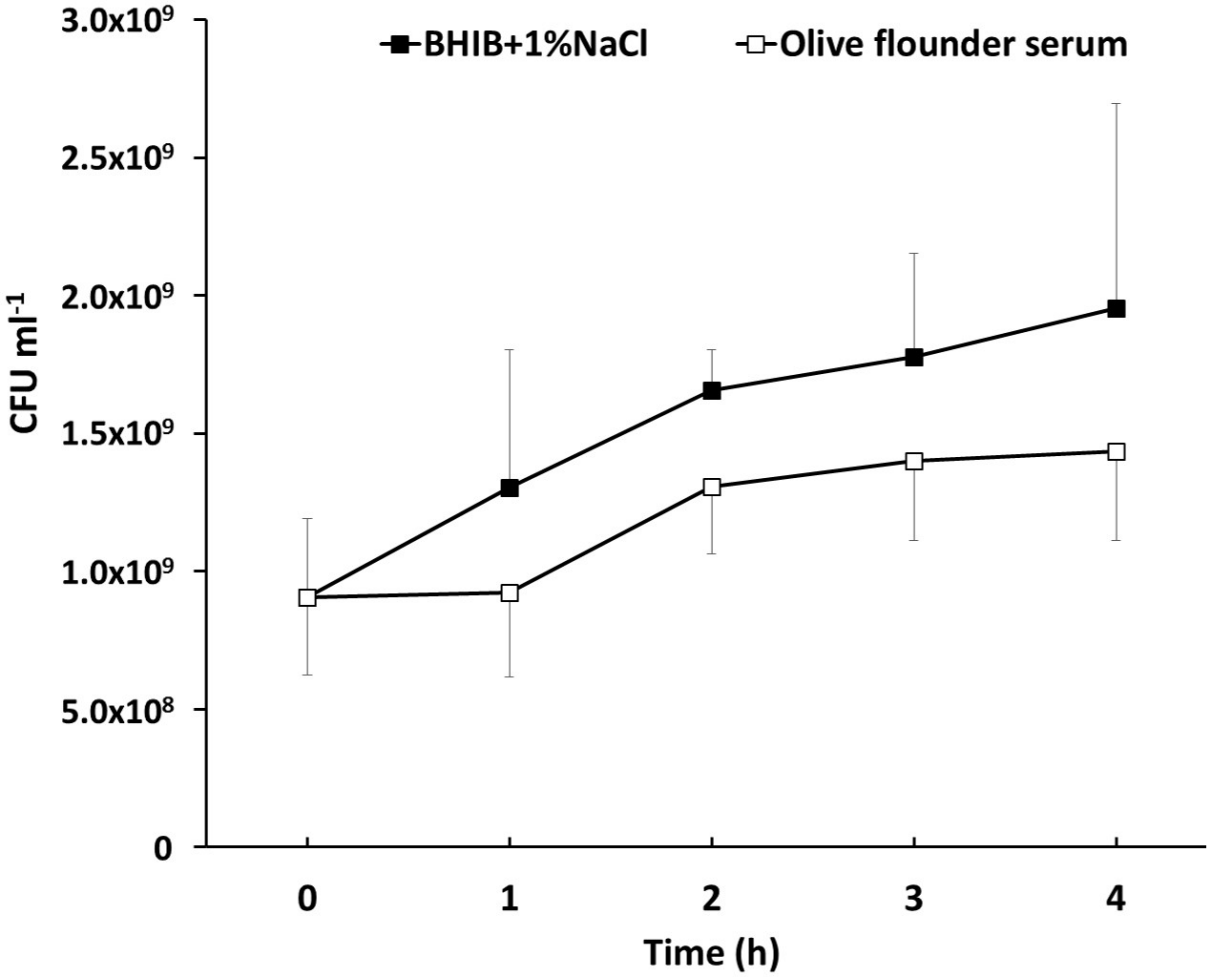
Bacterial growth in broth medium and olive flounder serum. Growth was measured every hour by plate counting during 4 hours of incubation. Error bars indicate standard deviation of the average of three biological replicates.

Bacterial growth in culture medium and fish serum is shown in Fig 1. While bacteria continued to grow in the culture medium (~ 2-fold increase at 4 hpe), the viability was maintained in the serum for 4 h. This result shows that *S*. *parauberis* SPOF3K is able to survive and persist in fish serum containing various antimicrobial components [17].

### Transcriptome data

**Fig 2:**
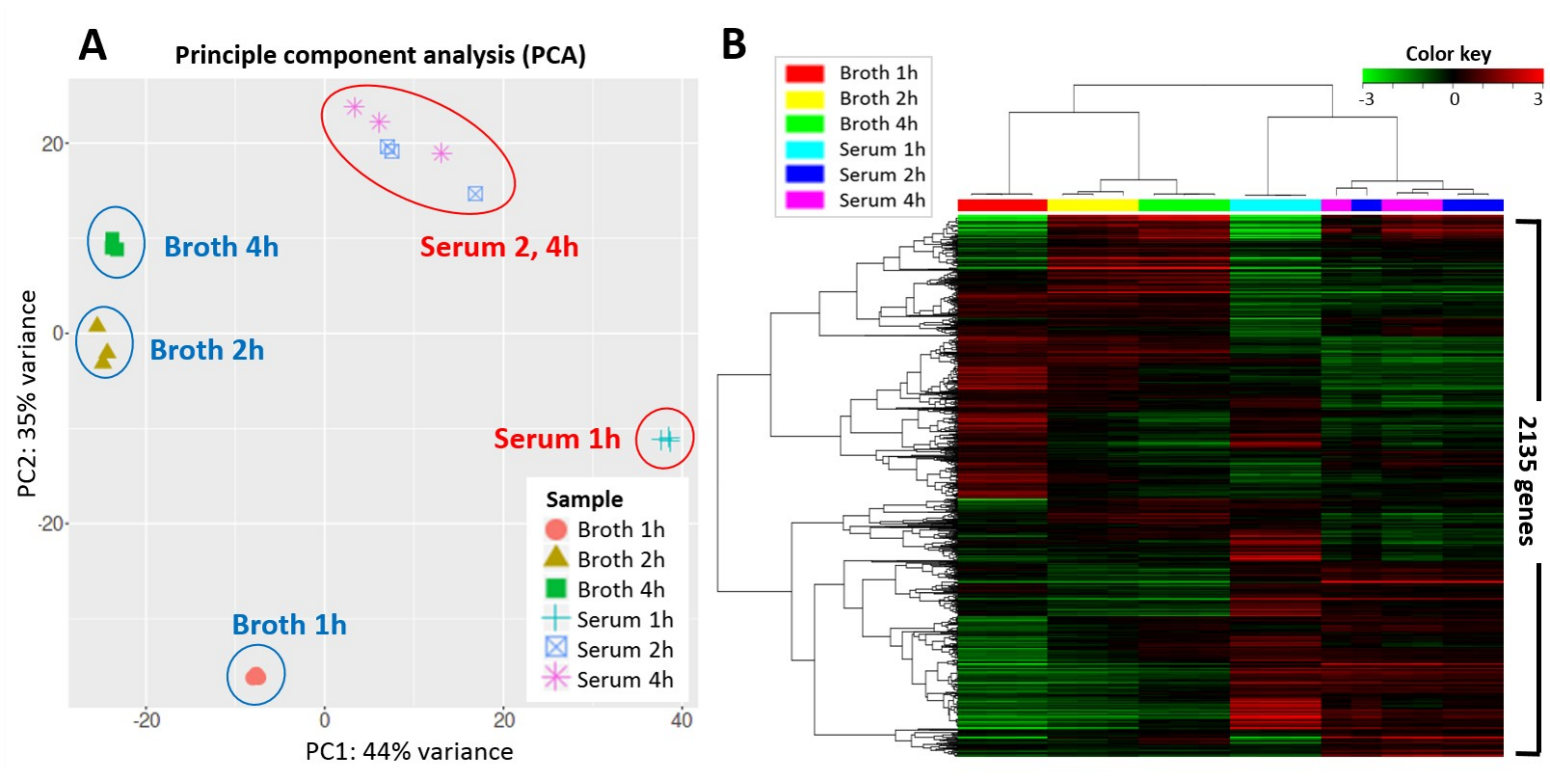
Transcriptome dynamics of *Streptococcus parauberis* SPOF3K in fish serum and broth medium. (A) Principle component analysis (PCA) of the SPOF3K transcriptome during culture in olive flounder serum and broth. Each shape represents an individual group. The PCA plot captures the variance in the dataset in terms of the principal components and displays the most significant of these on the x and y axes. The results indicate that the transcriptome data are of high quality, as the triplicate samples are clustered together according to incubation conditions; the 2 and 4 h serum samples were closely related in terms of their transcriptional patterns. (B) Hierarchical clustering heatmap of the expression profiles of all genes (2,135 genes). The colored bars above the heatmap indicate the individual groups. The color key indicates *z*-score and displays the relative expression levels: green, lowest expression; black, intermediate expression; red, highest expression.

**Fig 3:**
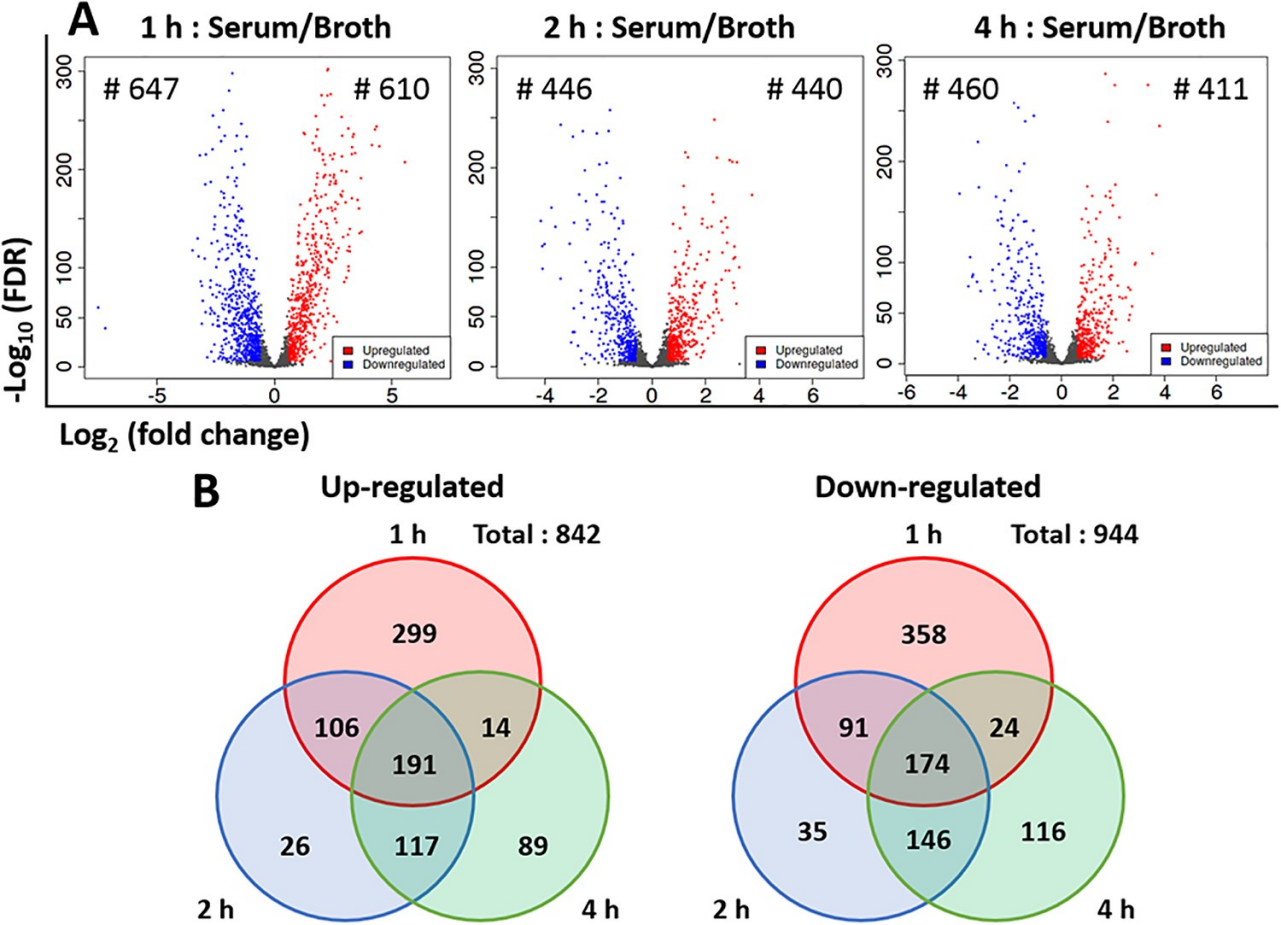
Differentially expressed genes. (A) Volcano plots show differences in gene expression in the serum-cultured vs. broth-cultured conditions. Colored circles indicate significantly up- (red) and down-regulated (blue) genes (FDR < 1e^-5^ and |fold change| > 1.5), which are also indicated by number (#). (B) Three-way Venn diagram illustrating the bacterial genes that were altered in the serum culture relative to the broth culture condition (1, 2 and 4 h). The findings indicate that 191 and 174 transcripts were consistently up- and down-regulated (FDR < 1e^-5^ and |fold change| > 1.5), respectively, in serum compared to medium.

RNAseq analysis generated an average of 25,249,899 reads in the respective samples (S1 Table). After removal of ribosomal RNA, intergenic reads, and low-quality reads through quality trimming, the final mapping rate of filtered transcript reads into the reference genome was 87.1–98.2% (S1 Table). The RNA sequencing data can be found in the NCBI sequence Read Archive (SRA) under accession number of PRJNA689581. PCA (Fig 2a) and a hierarchical clustering heatmap (Fig 2b) showed that the transcriptome data obtained in this study was well-clustered depending on culture condition and sampling time point. In addition, the transcriptome profiles of samples taken from the medium and serum at 2 and 4 h post-exposure (hpe) were more similar than those of samples collected at 1 hpe. This indicates that there was a systemic alteration after 1 h of exposure to each condition in terms of gene expression (Fig 2). According to the DGE analysis, 610 (28.5%), 440 (20.6%), and 411 (19.2%) genes were significantly up-regulated at 1, 2, and 4 hpe, respectively, in the fish serum compared to the culture medium, while 547 (25.6%), 446 (20.9%), and 460 (21.5%) genes were significantly down-regulated at 1, 2, and 4 hpe, respectively (Fig 3a). Three-way Venn diagram analysis of the identified DEGs showed that 191 (8.9%) and 174 (8.1%) genes were significantly up- and down- regulated, respectively, regardless of sampling time point (Fig 3b).

A plasmid of *S*. *parauberis* SPOF3K contains 13 genes [9]. Of these genes, *inlJ* (SPSF3K_02212) [25] and *tetS* (SPSF3K_02213) [26] have been implicated in the pathogen’s virulence and antibiotic resistance, respectively. Interestingly, all genes in this plasmid were significantly up-regulated in the presence of olive flounder serum at 1 hpe (Table 1). Genes responsible for plasmid replication (*repB*; SPSF3K_02216–17) and mobilization (*mobC*; SPSF3K_02219–21) showed the greatest DGE (fold change of 14.7–34.5) (Table 1). The Rep and Mob proteins are multifunctional elements that are essential for the initiation of both plasmid replication and conjugation in the rolling circle replication mechanism [27]. This result indicates that *S*. *parauberis* may increase its plasmid copy number when it encounters fish serum. Several previous studies [28–30] have demonstrated that plasmid copy number is correlated with bacterial virulence. For example, a previous study on a total of 32 *Bacillus anthracis* strains showed that strains harboring higher plasmid copy numbers tend to be more pathogenic to guinea pigs than those with lower copy numbers [28]. Also, animal- and human-derived *B*. *anthracis* isolates have higher plasmid copy numbers and are more pathogenic than those from environmental sources [30]. Plasmid copy number and the expression level of a plasmid replication-related gene, *repA*, in *Yersinia pseudotuberculosis* are increased in mice infected with the pathogen, while infectivity is reduced when the plasmid copy number is genetically restricted [29]. Taken together, our results indicate that this plasmid might be important for bacterial survival in fish serum at the very early stage of infection.

**Table 1:** SPOF3K plasmid gene expression.

Gene name	Gene locus	Descriptions	Log_2_ (Fold changes)		
			1 hpe	2 hpe	4 hpe
*inlJ*	SPSF3K_02212	Internalin J	0.78	-	-
*tetS*	SPSF3K_02213	Tetracycline resistance protein TetS	2.76	-	-
/	SPSF3K_02214	GNAT family N-acetyltransferase	3.30	-	-0.75
/	SPSF3K_02215	DUF536 domain-containing protein	3.95	-	-0.78
*repB*	SPSF3K_02216	RepB family plasmid replication initiator protein	3.88	-	-
*repB*	SPSF3K_02217	Replication initiator protein RepB	3.26	-	-
*/*	SPSF3K_02218	Fic family protein	4.28	1.64	0.67
/	SPSF3K_02219	Mobilization protein	4.47	1.70	0.71
/	SPSF3K_02220	Relaxase/mobilization nuclease domain containing protein	4.97	1.56	-
*mobC*	SPSF3K_02221	Plasmid mobilization relaxosome protein MobC	5.11	1.09	-
/	SPSF3K_02222	IS6 family transposase	0.93	-	-0.80
*/*	SPSF3K_02223	GNAT family N-acetyltransferase	2.06	0.67	-
/	SPSF3K_02224	Recombinase family protein	2.50	1.03	-

-, Not significant (|fold change| > 1.5 and FDR < 1e-5).

### Gene expression profiling based on functional categories

**Fig 4:**
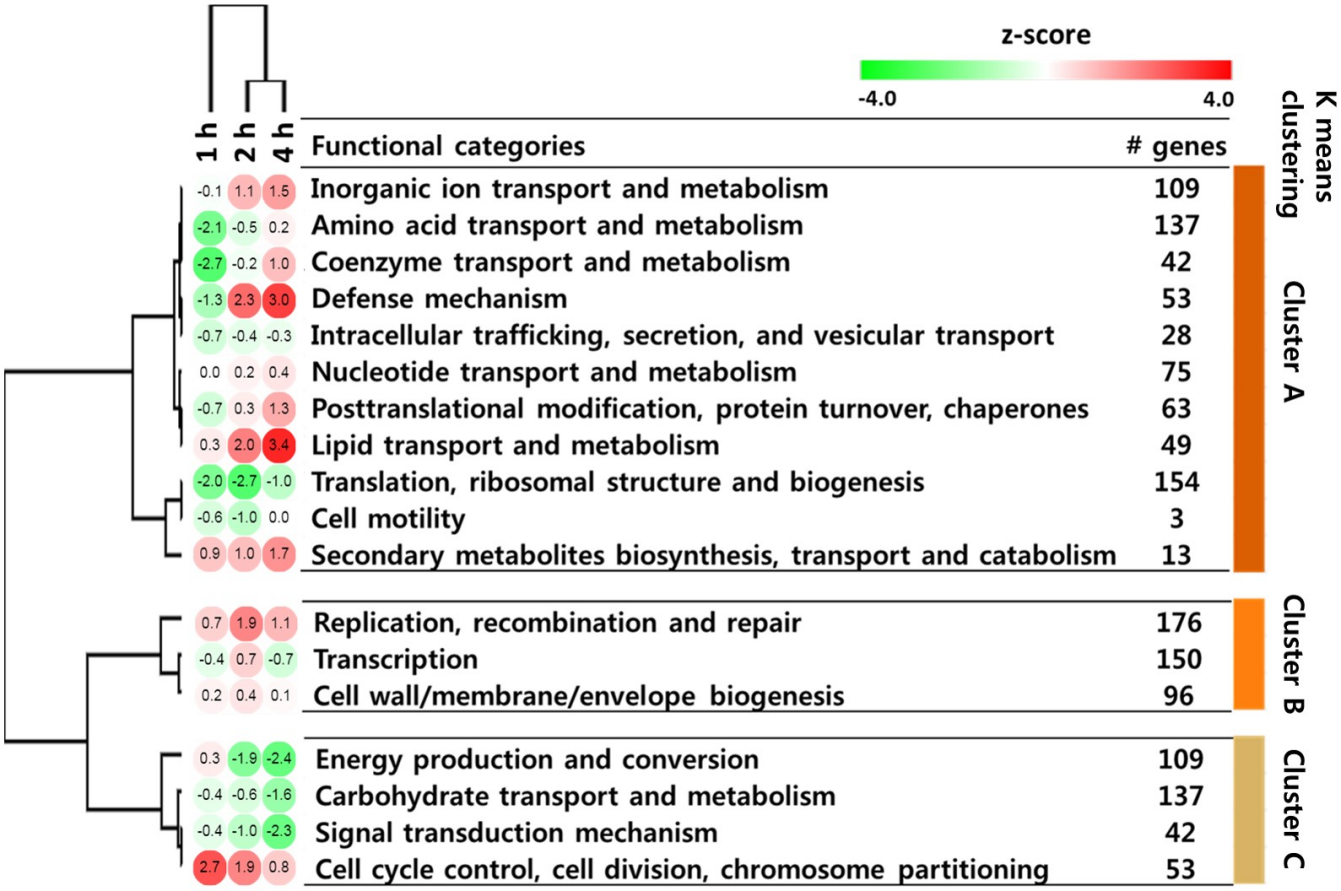
Gene expression profiling based on functional categories. Heatmap representing the expression dynamics of 18 functional categories in SPOF3K during incubation. A total of 1,432 genes were classified into 18 functional eggNOG categories (the unknown function category was excluded). The numbers in the heatmap indicate *z*-score (substitution of the number of significantly up- and down-regulated genes divided by the square of the total number of differentially expressed genes), and the colors display relative expression levels (*z*-score): green, lowest expression; red, highest expression. The colored bars on the right side of the figure represent the three clusters as determined by k-means clustering analysis, which correspond to the results of hierarchical analysis.

To understand the global transcriptome dynamics of *S*. *parauberis* grown in fish serum, the expression patterns of 1,432 genes in 18 functional categories were analyzed (Fig 4). As a result, three clusters were generated by hierarchical and k-means clustering analysis (*z*-score, shown in colored circle) according to sampling time point. In addition, the top twenty differentially regulated genes were listed with their functional categories and clusters in Table 2.

**Table 2:** The top twenty differentially expressed genes during incubation in fish serum.

Gene name	Gene locus	Descriptions	Log_2_ (Fold changes)			Functional categories*
			1hpe	2 hpe	4 hpe	
**Cluster A**						
*murM*	SPSF3K_00276	UDP-N-acetylmuramoylpentapeptide-lysine N(6)-alanyltransferase	3.1	3.0	3.5	V
*clpL*	SPSF3K_01110	Probable ATP-dependent Clp protease ATP-binding subunit	3.6	3.7	3.7	O
*dnaJ*	SPSF3K_02051	Chaperone protein DnaJ	-	4.2	4.5	O
*dnaK*	SPSF3K_02052	Chaperone protein DnaK	1.0	4.7	4.8	O
*grpE*	SPSF3K_02053	Protein GrpE	1.5	4.7	4.7	O
**Cluster B**						
/	SPSF3K_00044	DNA-binding transcriptional regulator, PadR family	6.9	3.2	-	K
/	SPSF3K_00093	DNA-binding transcriptional regulator, XRE-family	5.6	1.0	-	K
/	SPSF3K_00479	DNA-binding transcriptional regulator, MerR family	4.2	1.4	-	K
/	SPSF3K_00485	DNA-binding transcriptional regulator, PadR family	4.6	2.4	0.8	K
*hrcA*	SPSF3K_02054	Heat-inducible transcription repressor HrcA	1.5	4.6	4.4	K
*vanY*	SPSF3K_01228	Serine-type D-Ala-D-Ala carboxypeptidase	3.4	3.2	3.9	M
*dacC*	SPSF3K_00629	Serine-type D-Ala-D-Ala carboxypeptidase	3.8	0.8	-	M
*licR*	SPSF3K_00180	Probable licABCH operon regulator	-	-4.2	-3.5	K
*lrgB*	SPSF3K_00605	Antiholin-like protein LrgB	-7.5	-2.6	-2.8	M
**Cluster C**						
*nagB*	SPSF3K_01753	Glucosamine-6-phosphate deaminase	4.6	2.3	1.7	G
/	SPSF3K_02218	Fic family protein	4.3	1.6	0.7	D
*srlA*	SPSF3K_00182	Glucitol/sorbitol permease IIC component	-	-4.1	-3.5	G
*srlE*	SPSF3K_00183	Protein-N(pi)-phosphohistidine—sugar phosphotransferase	-	-4.1	-3.6	G
*srlB*	SPSF3K_00184	Protein-N(pi)-phosphohistidine—sugar phosphotransferase	-	-3.4	-3.3	G
*ptsG*	SPSF3K_00506	Protein-N(pi)-phosphohistidine—sugar phosphotransferase	-1.0	-3.1	-2.7	G

-, Not significant (|fold change| > 1.5 and FDR < 1e-5).

* eggNOG categories: V, Defense mechanism; O, Posttranslational modification, protein turnover, chaperones; K, Transcriptions; M, Cell wall/membrane/envelope biogenesis; G, Carbohydrate transport and metabolism; D, Cell cycle control, cell division, chromosome partitioning.

A total of 726 genes were divided into 11 functional categories. These genes were designated cluster A (Fig 4). The expression levels of the genes in this cluster became elevated as time went on. Genes in all the categories in this cluster may be essential for *S*. *parauberis* survival and proliferation, especially in fish serum, given the gradual increase in their expression levels during the incubation period. Defense mechanism was one of the most over-represented categories in cluster A (*z*-scores of 2.3 and 3.0 at 2 and 4 hpe, respectively). This finding coincides with the results of previous study [12] on *Staphylococcus aureus* cultured in human serum and blood. Some major components of the defense mechanism category include transport-related proteins (S2 Table). In many bacterial pathogens, activation of various substrate transporters has been shown to be essential for survival, as it maintains physiological balance in response to environmental changes as well as full pathogenicity [31]. In agreement with previous findings, genes encoding MsbA (SPSF3K_00195–6), CydCD (SPSF3K_00388–9), and FbpC (SPSF3K_00449), which are known to be responsible for lipid transport [32], aerobic respiration [33], and iron import [34], respectively, were significantly up-regulated in the present study (S2 Table).

Notably, *murM* (SPSF3K_00276) was significantly up-regulated (~ 8.4-, 8.2-, and 11.4-fold at 1, 2 and 4 hpe, respectively, Table 2 and S2 Table). MurM participates in streptococcal cell wall formation via addition of _L_-Ser- _L_-alanine or _L_-Ala-_L_-Ala to form peptide inter-bridges between peptidoglycan precursor units [35]. A mutant strain of *Streptococcus pneumoniae* with a *murM* gene deletion showed increased sensitivity to lysozyme [36]. The significant increase in expression of *S*. *parauberis murM* observed in this study might confer greater resistance against immunological components of the serum (e.g., lysozyme). Another enzyme that mediates lysozyme resistance, OatA (SPSF3K_01374), which encodes peptidoglycan O-acetyltransferase, was significantly up-regulated at 1 and 2 hpe (~ 2-, and 1.6-fold, respectively) (Table 3). OatA modifies the C6-OH group of muramic acid through O-acetylation, providing bacteria with an increased degree of lysozyme resistance [37]. In addition, genes encoding serine-type D-Ala-D-Ala carboxypeptidase DacC (SPSF3K_00629) and VanY (SPSF3K_01228) were remarkably up-regulated in the presence of fish serum (~ 10-fold change at 1 hpe) (Table 2). This enzyme, one of penicillin binding proteins (PBPs), is involved in the last step of peptidoglycan biosynthesis, which is critical for maintaining bacterial structural integrity, resistance to environmental changes, and full virulence [38, 39]. Additional PBP-encoding genes, such as *pbp1A* (SPSF3K_01971), *pbp1B* (SPSF3K_00396) and *ampC* (SPSF3K_02201), were also found to be up-regulated in the fish serum (~ 2.4-, ~ 4.8- and ~ 3.9-fold changes respectively at 1 hpe) (S1 File). Taken together, the overexpression of these enzymes indicates that *S*. *parauberis* can modify its peptidoglycan layer to resist against antimicrobial activities in olive flounder serum.

**Table 3:** Changes in expression level of major virulence-related genes.

Gene name	Gene locus	Description	Log_2_ (Fold changes)		
			1 hpe	2 hpe	4 hpe
**Capsule**					
*hasA*	SPSF3K_00187	Hyaluronan synthase	-	0.79	1.00
*hasB*	SPSF3K_00188	UDP-glucose 6-dehydrogenase	0.61	0.73	0.75
**Protease**					
*htrA/degP*	SPSF3K_00002	Serine protease	1.15	-	-0.91
*scp*	SPSF3K_00379	C5a peptidase	1.05	0.90	1.14
/	SPSF3K_01846	Lactocepin	1.68	0.97	1.47
**Adherence**					
*/*	SPSF3K_00426	Antiphagocytic M protein	-1.00	-	-
*lmb*	SPSF3K_01304	Laminin-binding protein	-1.83	-	-
*fbpA*	SPSF3K_01632	Fibronectin-binding protein	0.63	-	-
*inlJ*	SPSF3K_02212	Internalin J	0.78	-	-
**Enzyme**					
*gapA*	SPSF3K_00249	Glyceraldehyde 3-phosphate dehydrogenase (GAPDH)	0.91	-	0.59
*ef-tu*	SPSF3K_00907	Elongation factor thermo-unstable	0.69	0.94	0.64
*lgt*	SPSF3K_00871	Prolipoprotein diacylglyceryl transferase	1.22	0.60	-
*lspA*	SPSF3K_01057	Signal peptidase II	-1.88	-	-
*eno*	SPSF3K_00967	Streptococcal enolase	-	-0.59	-0.63
**Iron uptake**					
*rgpD*	SPSF3K_00008	Putative polysaccharide ABC transporter, ATP-binding protein	2.21	0.94	-
*psaA*	SPSF3K_00778	Putative metal binding protein of ABC transporter (lipoprotein)	1.34	-0.64	-
**Cell wall associated**					
*srtA*	SPSF3K_01301	Sortase A	1.99	1.18	0.58
*oatA*	SPSF3K_01374	Peptidoglycan O-acetyltransferase	0.97	0.63	-
**Others**					
*mf2*	SPSF3K_00129	Mitogenic factor 2	2.30	1.88	1.89
*bcsA*	SPSF3K_02074	Cellulose synthase (UDP-forming)	-1.05	0.97	0.95
*adrA*	SPSF3K_02080	Diguanylate cyclase/phosphodiesterase	-3.10	1.10	1.13

-, Not significant (|fold change| > 1.5 and FDR < 1e-5).

In cluster B, genes encoding DNA-binding transcriptional regulators of PadR (SPSF3K_00044 and SPSF3K_00485), XRE (SPSF3K_00093) and MerR (SPSF3K_00479) families were highly up-regulated in fish serum (~17–120-fold change at 1 hpe) (Table 2). Previous studies [40–42] have demonstrated that these transcriptional regulator families involve in tolerance against environmental stress, antibiotic resistance as well as full pathogenicity by regulating gene expression harboring their respective promotors.

Cluster C is composed of four functional categories including 341 genes. The expression levels of these genes decreased as time went by. Of these four, the categories of energy production and conversion and carbohydrate transport and metabolism sampled were significantly under-represented at 4 hpe. In the carbohydrate transport and metabolism category, the main genes identified were phosphotransferase system (PTS)-related genes responsible for various carbohydrate transport mechanisms; these were significantly down-regulated (Table 2 and S3 Table). The phosphorylation status of the PTS reflects the availability of carbohydrates as well as bacterial energy conditions [43]. In the SPOF3K genome, 33 genes involved in the PTS which target 12 carbohydrates were identified (KEGG KO number: ko02060) [44]. The majority of these genes were significantly down-regulated after four hours of exposure to fish serum (Table 2 and S3 Table). For instance, a gene encoding PtsG (SPSF3K_00506), a glucose-specific PTS component, was down-regulated (~ 1.9-, 8.5-, and 6.4-fold change at 1, 2 and 4 h, respectively) in the presence of fish serum (Table 2 and S3 Table). The under-representation of these genes might be due to the limited availability of carbohydrates in fish serum compared to nutrient-enriched culture medium.

### Stress response in olive flounder serum

Induction of stress responses often represents the bacterial response to environmental changes, specifically adaptation to the host [45]. In addition to playing a key role in protein folding, heat-shock proteins (Hsp) regulate transcription of various virulence-related factors in many bacterial species including those in the genus *Streptococcus* [46–48]. Remarkably, *groESL* (SPSF3K_00141–142) and *dnaK-dnaJ-grpE* (SPSF3K_002050–2052) chaperone (also known as Hsp60 and Hsp70) and their regulatory genes *ctsR* (SPSF3K_00139) and *hrcA* (SPSF3K_02054) were all significantly up-regulated at all sampling time points, showing increasing fold changes as time went on (~ 2-fold change at 1 hpe and ~ 7–29-fold change at 2 and 4 hpe) (Tables 2 and 4). These chaperones can refold, disaggregate, and properly assemble abnormal or misfolded polypeptides resulting from cell damage induced by a hostile environment [45]. This indicates that bacteria incubated in fish serum are under severe stress due to the antibacterial activity of various components of the serum. Therefore, these proteins may play an essential role in maintaining cellular structure and integrity.

**Table 4:** Significant changes in expression of stress-related genes.

Gene name	Gene locus	Descriptions	Log_2_ (Fold changes)		
			1 hpe	2 hpe	4 hpe
***DnaK* operon**					
*dnaJ*	SPSF3K_02051	Chaperone protein DnaJ	-	4.17	4.47
*dnaK*	SPSF3K_02052	Chaperone protein DnaK	1.02	4.67	4.84
*grpE*	SPSF3K_02053	Heat shock protein GrpE	1.53	4.75	4.73
***GroEL* operon**					
*groES*	SPSF3K_00141	10 kDa chaperonin	1.10	2.81	3.79
*groEL*	SPSF3K_00142	60 kDa chaperonin	1.31	2.90	3.34
***Clp* protease family**					
*clpC*	SPSF3K_00140	ATP-dependent Clp protease ATP-binding subunit	1.72	1.87	2.08
*clpE*	SPSF3K_01798	ATP-dependent Clp protease ATP-binding subunit	-	3.21	3.47
*clpL*	SPSF3K_01110	Probable ATP-dependent Clp protease ATP-binding subunit	3.61	3.73	3.66
*clpP*	SPSF3K_00735	Endopeptidase Clp	-1.50	-	0.58
*clpX*	SPSF3K_01107	ATP-dependent Clp protease ATP-binding subunit	-1.80	-1.25	-0.7
**Regulatory genes**					
*hrcA*	SPSF3K_02054	Heat-inducible transcription repressor HrcA	1.48	4.60	4.41
*ctsR*	SPSF3K_00139	Transcriptional regulator CtsR	2.18	1.81	1.90

-, Not significant (|fold change| > 1.5 and FDR < 1e-5).

In addition, several genes encoding Clp chaperone proteins were significantly up-regulated by serum treatment (Table 4). This multigene chaperone family is able to efficiently disaggregate and refold a variety of protein aggregates [49]. Several previous studies have shown that Clp proteases are essential for bacterial stress tolerance and full virulence in the host [50, 51]. Three Clp ATPase-encoding genes, *clp*C (SPSF3K_00140), *clpL* (SPSF3K_01110) and *clpE* (SPSF3K_01798), were significantly up-regulated at 2 and 4 hpe (~ 4–13-fold change), while another Clp ATPase-encoding gene, *clpX* (SPSF3K_01107), and an endopeptidase-encoding gene, *clpP* (SPSF3K_00735), were not significantly up-regulated (Tables 2 and 4). Previous studies [49, 51] demonstrated that three ATPases, ClpC, ClpE, and ClpX, can recognize and bind ClpP protease to form a complex (e.g., ClpXP) that can cleave misfolded proteins. In contrast, streptococcal ClpL does not require any co-chaperones to exhibit proteolytic activity [51, 52]. Therefore, ClpL seems to be more crucial to the *S*. *parauberis* stress response against fish serum than the other ClpP-dependent proteases. However, further experimental studies are needed to better understand the mode of action of Clp protease family members in *S*. *parauberis*.

### Differentially expressed virulence-related genes

Hyaluronic acid capsule (M protein) and capsular polysaccharide (CPS) are major virulence factors in several streptococcal species; they can increase cell adhesion ability and reduce the phagocytic and opsonic activities of the host [53–55]. In this study, *hasA* (SPSF3K_00187), a gene encoding hyaluronan synthase, and *hasB* (SPSF3K_00188), a gene encoding UDP-glucose dehydrogenase, were significantly up-regulated in serum-incubated samples, although *hasC* (SPSF3K_00192), a glucose-1-phosphate uridyltransferase gene, did not show significant transcriptional changes during incubation in serum (Table 3). Similarly, a previous study [56] demonstrated that inactivation of *hasA* and *hasB* results in a significant decrease in encapsulation in group A Streptococcus (GAS), while inactivation of *hasC* does not affect the GAS encapsulation level. Ashbaugh et al. [56] suggested that enzymes other than HasC can produce UDP-glucose, a precursor of hyaluronic acid. A recent study [57] showed that UDP-glucose can be metabolically synthesized through the galactose pathway with the involvement of GalE, a UDP-glucose 4-epimerase-encoding gene, and GalT, a galactose-1-phosphate uridylyltransferase. Our strain harbors *galT* (SPSF3K_00522) and *galE* (SPSF3K_00523), which seem to be responsible for the production of UDP-glucose. These two genes were significantly up-regulated in fish serum, with very similar expression patterns at 1 and 2 hpe (~ 3- and 1.5-fold change, respectively). Antiphagocytic M protein (SPSF3K_00426) showed significant down-regulation at 1 hpe (Table 3), in agreement with the results of a previous study [58], showing that hyaluronic acid capsule rather than M protein plays a crucial role in *Streptococcus pyogenes* survival in human serum. In this study, there were no significant differences in the expression of CPS operon (nineteen genes (SPSF3K_01554–1572)) between *S*. *parauberis* incubated in serum and culture medium (S1 File). These results indicate that the hyaluronic acid capsule of *S*. *parauberis* SPO3K may play an important role in its resistance against fish serum.

The Sortase A**-**encoding gene *srtA* (SPSF3K_01301) was up-regulated at all sampling time points (~ 4-, 2.3-, and 1.5-fold change at 1, 2, and 4 hpe, respectively) (Table 3). Sortase A is a membrane-associated transpeptidase responsible for covalent anchoring of many virulence factors of Gram-positive bacteria to cell wall peptidoglycan by recognizing and cleaving a signal peptide containing a C-terminal LPXTG (or LPXTA) motif [59]. Several studies have shown that inactivation of *srtA* can result in significant attenuation of bacterial pathogenicity (e.g. in the closely related bacterial fish pathogen *Streptococcus iniae*) [60]. Conversely, over-expression of *srtA* indicates enrichment of bacterial surface proteins in cell walls. Ten CDSs containing LPXTG (or LPXTA) motifs were found in the SPOF3K genome using a hidden Markov model (HMM) (S4 Table) [61]. These include M-like protein (SPSF3K_00426), C5a peptidase (SPSF3K_00380), and Internalin J (SPSF3K_02222), which are sortase-mediated cell wall-anchored proteins and virulence factors. C5a peptidase can specifically cleave complement component 5a (C5a) [62] and was significantly over-expressed in all serum samples (~ 2-fold changes) (Table 3 and S4 Table). The increase in C5a peptidase in the cell wall of *S*. *parauberis* in this study may correspond to a significant reduction in the serum level of C5a, which is an important chemoattractant molecule involved in innate immunity [17], such that the bacterium has a better chance to survive and multiply in the host.

GAPDH (SPSF3K_00249) and an elongation factor (Ef-Tu; SPSF3K_00907), which have multiple biological functions, were up-regulated in fish serum at 1 and 4 hpe (~ 2 and 1.5-fold changes respectively) (Table 3). The release of GAPDH in group B Streptococcus (GBS) can cause re-association of bacterial cells, stimulate host IL-10 production (thus further impairing neutrophil recruitment), and induce macrophage apoptosis [63]. Besides its role in translation as a GTPase, Ef-Tu is also involved in pathogenesis as it can serve as an adhesin and an immune evasion factor by binding to complement regulatory factors such as factor H, which further inactivates complement C3b [64]. Increased expression of these multifunctional genes in fish serum-exposed *S*. *parauberis* implicates them in *S*. *parauberis* pathogenesis.

Bacterial lipoproteins have a variety of physiological functions, such as nutrient acquisition, adaptation to environmental changes, protein maturation, and adherence [65]. In Gram-positive bacteria, pro-lipoprotein diacylglyceryl transferase (Lgt) (SPSF3K_00871) and signal peptidase II (LspA) (SPSF3K_01057) are known to be involved in maturation of bacterial lipoproteins. In this study, we found that the expression of *lgt* was significantly up-regulated at 1 and 2 hpe (~ 2.3- and 1.5-fold change, respectively) (Table 3). This indicates that bacterial lipoproteins are synthesized and mature after exposure to fish serum. In fact, a number of lipoproteins that work as substrate-binding proteins for ABC transporters are essential for bacterial survival as they maintain physiological balance in a hostile environment and sustain virulence throughout pathogen-host interactions [31]. Several significantly up-regulated ABC transporter-related genes were identified (KEGG KO number: ko02010, Table 5). These include the biotin transporter-encoding genes *bioY* (SPSF3K_00173–4), *ecfT* (SPSF3K_00021 and SPSF3K_00582), and *ecfA1* (SPSF3K_00583), the osmoprotectant transporters-encoding genes, *proVWX* (SPSF3K_00166–7) and *opuC* (SPSF3K_01282), as well as the oligopeptide permease transport system-encoding *opp* operon (SPSF3K_01238–41). Many previous studies demonstrated that these genes are closely related to survival and virulence in several bacterial species under stressful conditions as well as in host species. For example, the significant up-regulation of osmoprotectant transporter-related genes indicates that they are important for counterbalancing against osmotic stress, as previously described [66]. In addition, although *opp* operon activity is related to nutrient intake, it is also essential for bacterial pathogenicity, cytoadherence, and environmental adaptation in many different bacterial species [67].

**Table 5:** Significant changes in expression of ATP-binding cassette transporters.

Gene	Locus	Log_2_ (Fold changes)			K number
		1 hpe	2 hpe	4 hpe	
**Biotin**					
*bioY*	SPSF3K_00173	3.22	3.10	2.86	K03523
*bioY*	SPSF3K_00174	3.11	3.04	2.84	K03523
*ecfT*	SPSF3K_00021	-	0.86	-	K16785
*ecfT*	SPSF3K_00582	1.68	0.71	-	K16785
*ecfA1*	SPSF3K_00583	2.19	0.83	-	K16786
**Multidrug resistance / Hemolysin**					
*cylA*	SPSF3K_00217	1.10	0.66	-	K11050
*cylB*	SPSF3K_00218	1.34	0.75	0.76	K11051
**ABCC Subfamily**					
*cydC*	SPSF3K_00388	-	0.69	-	K16013
*cydD*	SPSF3K_00389	-	0.74	0.68	K16012
ABCC-BAC	SPSF3K_00478	3.16	1.24	-	K06148
CFTR, ABCC7	SPSF3K_01680	-1.03	-	-	K05031
**Iron complex**					
ABC.FEV.A	SPSF3K_00008	2.21	0.94	-	K02013
ABC.FEV.P	SPSF3K_00728	-	0.71	1.04	K02015
ABC.FEV.P	SPSF3K_00729	-	-	0.85	K02015
**Glycine betaine/proline**					
*proV*	SPSF3K_00166	1.54	0.60	-	K02000
*proX proW*	SPSF3K_00167	1.23	0.75	0.58	K02001-2
**Osmoprotectant**					
*opuC*	SPSF3K_01282	1.65	1.19	1.22	K05845
**Oligopeptide**					
*oppA*	SPSF3K_00630	1.72	-	-	K15580
*oppB*	SPSF3K_00631	1.77	-	-	K15581
*oppC*	SPSF3K_00632	1.40	-	-	K15582
*oppD*	SPSF3K_00633	1.35	-	-	K15583
*oppF*	SPSF3K_00634	1.28	-	-	K10823

-, Not significant (|fold change| > 1.5 and FDR < 1e-5).

In the SPOF3K genome, we identified a seven-gene cluster including BC synthase (*bcsA*; SPSF3K_02074), PNAG synthase (*pga*; SPSF3K_02077), and diguanylate synthase/phosphodiesterase (*dgc/pdeA*; SPSF3K_02080), which is known to modulate the concentration of cyclic-di-GMP, a positive regulator of bacterial cellulose (BC) synthesis [68]. Bacterial biofilm is formed with extracellular polymeric substances (EPS). BC is one of the major components of EPS [69]. In addition to BC, poly-N-acetylglucosamine (PNAG) is another well-known polysaccharide component of EPS. It is an adhesion molecule that is required for microbial biofilm formation in pathogenic bacterial species [70]. All of those biofilm-related genes were significantly up-regulated at 2 and 4 hpe in *S*. *parauberis* incubated in fish serum (Table 3). França and Cerca [71] reported that the transcription levels of biofilm-related genes including *icaA*, *irgB*, and *capA* in *Staphylococcus epidermidis* significantly increase when the bacterium is incubated with human blood and plasma rather than culture medium. Those authors also suggested that various proteins in plasma may interact with bacterial cells to modulate the transcriptional levels of these genes. In accordance with this idea, our results indicate that this gene cluster may also be important for bacterial biofilm formation.

## Conclusions

**Fig 5:**
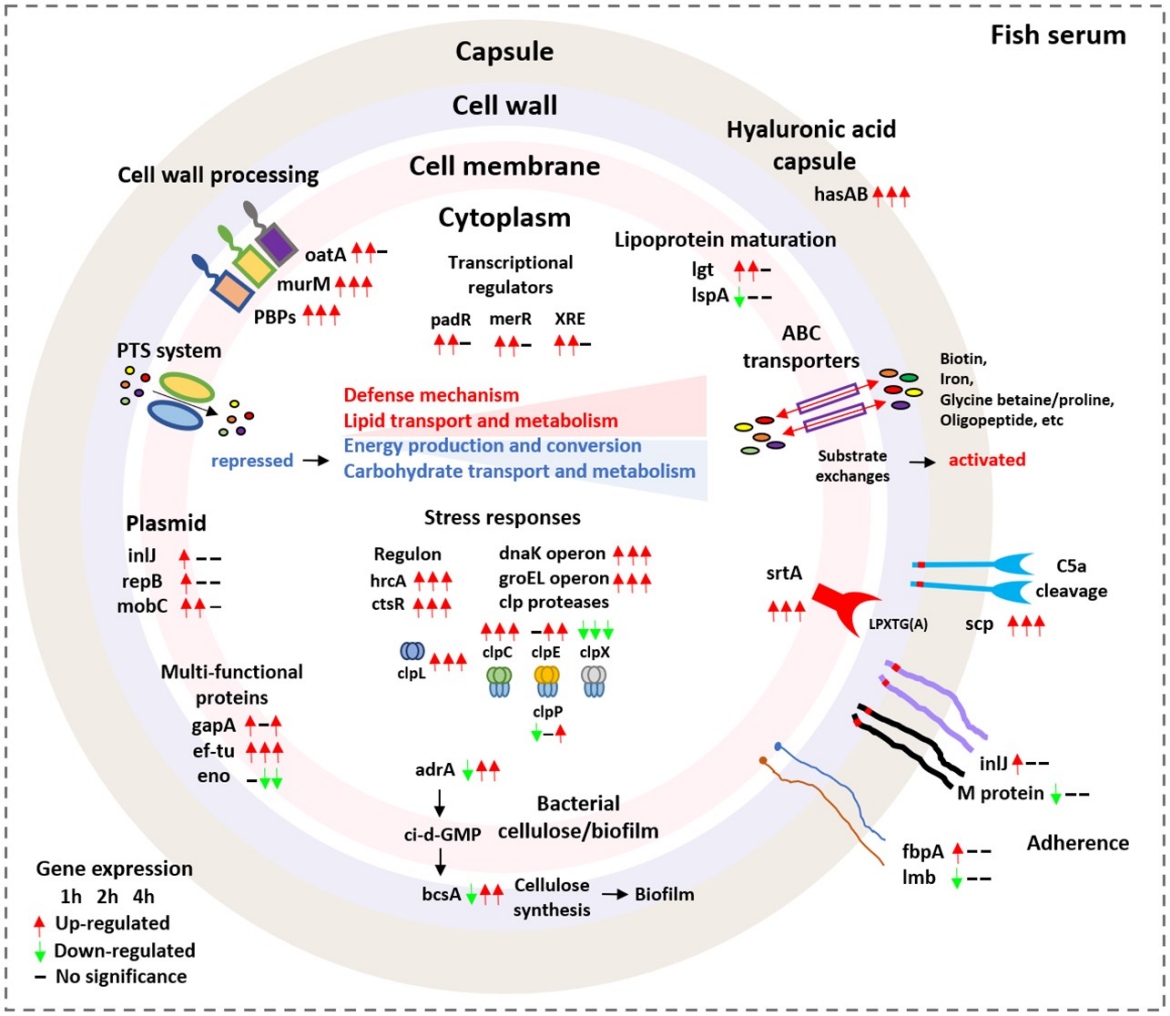
Schematic illustration of the transcriptional responses of *Streptococcus parauberis* SPOF3K after exposure to olive flounder serum. The schematic shows the proposed model of *S*. *parauberis* gene expression patterns involved in adaptation to olive flounder serum. Gene expression levels at each sampling time point (1, 2 and 4 h) are represented by red (up-regulated) and green (down-regulated) arrows and dashes (not significant).

In this study, we successfully explored the global transcriptomic dynamics of *S*. *parauberis* after exposure to olive flounder serum. Extensive remodeling of the bacterial transcriptome was identified in fish serum, which represents the early steps of *S*. *parauberis* adaptation in the host. The major genetic changes induced by exposure to fish serum include increased expression of stress resistance-related genes (e.g., *groEL*, *dnaK* operons and *clp* protease family genes), genes responsible for resistance to innate immunity in fish serum (e.g., *hasAB*, s*rtA*, *scp*, *oatA*, etc.) and important substrate transporters, which may be key factors contributing to *S*. *parauberis* survival in the host (Fig 5). The data presented here provide fundamental background knowledge that will aid in future studies on pathogenesis and help to identify putative targets for development of new diagnostic and prophylactic strategies.

## Supporting information

S1 Fig Pre-processing of transcriptomic data. (A) Distribution of total read counts (in millions), (B) Distribution of transformed (normalized) data and (C) Density plot of transformed data.
(TIF)

S1 Table Summary sequencing statistics of RNAseq. (DOCX)

S2 Table Expression level changes in defense mechanism category. (DOCX)

S3 Table Expression of genes involved in Phosphotransferase System (PTS). (DOCX)

S4 Table Gene expression of sortase A-mediated surface-anchored proteins containing LPXTG (or LPXTA) motif in C-terminal. (DOCX)

S1 File Differential gene expression data including raw read counts, normalized read counts and gene annotation. (XLSX)
