# Supplementary figures and images for: Transcriptome analysis unveils survival strategies of Streptococcus parauberis against fish serum
Source: PLoS One. 2021 May 26;16(5):e0252200. doi: 10.1371/journal.pone.0252200 (PMC8153452; doi:10.1371/journal.pone.0252200)

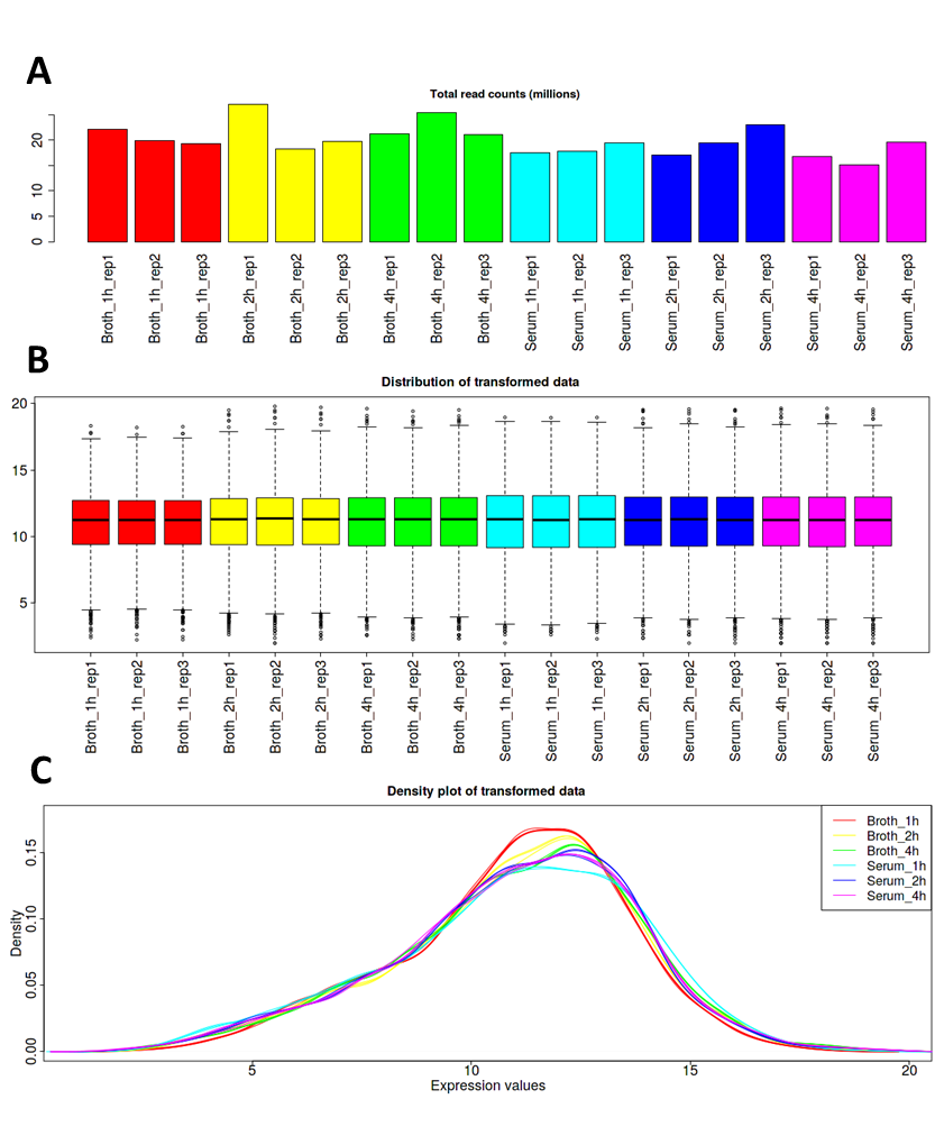

Supplement: S1 Fig — (A) Distribution of total read counts (in millions), (B) Distribution of transformed (normalized) data and (C) Density plot of transformed data. (TIF) [file pone.0252200.s001.tif]
