# Supplementary material for: Transcriptome analysis unveils survival strategies of Streptococcus parauberis against fish serum
Source: PLoS One. 2021 May 26;16(5):e0252200. doi: 10.1371/journal.pone.0252200 (PMC8153452; doi:10.1371/journal.pone.0252200)
Supplement: S1 Table — (DOCX) [file pone.0252200.s002.docx]

**S1 Table. Summary sequencing statistics**

| **Sample** | **Replication** | **Total reads** | **QC dropped reads** | **rRNA reads** | **Intergenic reads** | **Unmapped reads** | **mRNA reads** | **Mapping rate (%)** |
| --- | --- | --- | --- | --- | --- | --- | --- | --- |
| **1h Broth** | Rep 1 | 26,770,092 | 32,867 | 82,679 | 3,946,011 | 509,151 | 22,199,384 | 98.1 |
|  | Rep 2 | 24,374,137 | 25,214 | 70,082 | 3,854,583 | 581,570 | 19,842,688 | 97.6 |
|  | Rep 3 | 23,179,668 | 24,232 | 59,893 | 3,457,539 | 348,692 | 19,289,312 | 98.5 |
| **1h Serum** | Rep 1 | 25,251,382 | 37,280 | 1,362,287 | 2,921,966 | 3,381,741 | 17,548,108 | 86.6 |
|  | Rep 2 | 24,401,998 | 53,251 | 125,402 | 2,958,888 | 3,488,787 | 17,775,670 | 85.7 |
|  | Rep 3 | 26,812,486 | 75,319 | 114,781 | 3,322,495 | 3,820,511 | 19,479,380 | 85.8 |
| **2h Broth** | Rep 1 | 32,704,855 | 72,757 | 185,582 | 4,868,214 | 586,550 | 26,991,752 | 98.2 |
|  | Rep 2 | 22,241,039 | 24,044 | 43,744 | 3,351,331 | 512,304 | 18,309,616 | 97.7 |
|  | Rep 3 | 23,988,867 | 22,323 | 98,400 | 3,590,872 | 464,968 | 19,812,304 | 98.1 |
| **2h Serum** | Rep 1 | 21,681,056 | 35,977 | 46,249 | 3,001,294 | 1,533,612 | 17,063,924 | 92.9 |
|  | Rep 2 | 25,192,992 | 31,933 | 114,574 | 3,388,237 | 2,189,204 | 19,469,044 | 91.3 |
|  | Rep 3 | 28,994,847 | 30,108 | 164,176 | 3,834,481 | 1,972,758 | 22,993,324 | 93.2 |
| **4h Broth** | Rep 1 | 25,914,973 | 35,043 | 70,172 | 3,882,064 | 693,122 | 21,234,572 | 97.3 |
|  | Rep 2 | 31,124,601 | 45,938 | 66,256 | 4,809,186 | 834,349 | 25,368,872 | 97.3 |
|  | Rep 3 | 25,662,496 | 27,079 | 49,250 | 3,808,651 | 676,916 | 21,100,600 | 97.4 |
| **4h Serum** | Rep 1 | 21,280,621 | 21,749 | 38,338 | 3,133,132 | 1,327,861 | 16,759,541 | 93.8 |
|  | Rep 2 | 19,780,737 | 27,379 | 134,334 | 2,918,480 | 1,621,407 | 15,079,137 | 91.8 |
|  | Rep 3 | 25,141,336 | 24,005 | 153,175 | 3,591,375 | 1,815,921 | 19,556,860 | 92.8 |
