# Supplementary material for: Transcriptome analysis unveils survival strategies of Streptococcus parauberis against fish serum
Source: PLoS One. 2021 May 26;16(5):e0252200. doi: 10.1371/journal.pone.0252200 (PMC8153452; doi:10.1371/journal.pone.0252200)
Supplement: S2 Table — (DOCX) [file pone.0252200.s003.docx]

**S2 Table. Expression level changes in defense mechanism category**

| **Locus** | **Function of product** | **Log_2_ (Fold changes)** | | |
| --- | --- | --- | --- | --- |
|  |  | **1 hpe** | **2 hpe** | **4 hpe** |
| SPSF3K_00195 | Lipid A export ATP-binding/permease protein MsbA | 0.66 | 1.35 | 1.85 |
| SPSF3K_00196 | Lipid A export ATP-binding/permease protein MsbA | - | 1.18 | 1.83 |
| SPSF3K_00218 | hypothetical protein | 1.34 | 0.75 | 0.76 |
| SPSF3K_00276 | UDP-N-acetylmuramoylpentapeptide-lysine N(6)-alanyltransferase MurM | 3.07 | 3.03 | 3.51 |
| SPSF3K_00321 | Serine-type D-Ala-D-Ala carboxypeptidase | - | 0.76 | 0.86 |
| SPSF3K_00388 | ATP-binding/permease protein CydC | - | 0.69 | - |
| SPSF3K_00389 | ATP-binding/permease protein CydD | - | 0.74 | 0.68 |
| SPSF3K_00429 | Type I site-specific deoxyribonuclease | -2.73 | - | 0.70 |
| SPSF3K_00446 | Fe(3+) ions import ATP-binding protein FbpC | - | 0.62 | 0.76 |
| SPSF3K_00449 | ABC transporter G family member | - | 1.08 | 0.88 |
| SPSF3K_00473 | hypothetical protein | 1.24 | 1.20 | - |
| SPSF3K_00476 | hypothetical protein | 2.65 | 1.10 | - |
| SPSF3K_00478 | Ribose import ATP-binding protein RbsA | 3.16 | 1.24 | - |
| SPSF3K_01011 | Putative hemin transport system permease protein HrtB | -0.88 | - | 0.77 |
| SPSF3K_01385 | ABC transporter permease protein YxdM | - | 0.65 | - |
| SPSF3K_01447 | hypothetical protein | -1.01 | 0.74 | 0.99 |
| SPSF3K_01755 | hypothetical protein | - | 1.04 | 1.18 |
| SPSF3K_01847 | 5-methylcytosine-specific restriction enzyme | 2.27 | - | - |
| SPSF3K_01880 | Methionine import ATP-binding protein MetN | - | - | 0.61 |
| SPSF3K_01918 | Inner membrane transport permease | 1.00 | 1.05 | 1.34 |
| SPSF3K_02131 | Probable multidrug resistance protein NorM | - | 0.82 | 1.12 |
| SPSF3K_02201 | Beta-lactamase | 1.96 | 0.68 | - |

-, Not significant (|fold change| > 1.5 and FDR < 1e-5).
