# Supplementary material for: Transcriptome analysis unveils survival strategies of Streptococcus parauberis against fish serum
Source: PLoS One. 2021 May 26;16(5):e0252200. doi: 10.1371/journal.pone.0252200 (PMC8153452; doi:10.1371/journal.pone.0252200)
Supplement: S3 Table — (DOCX) [file pone.0252200.s004.docx]

**S3 Table. Expression of genes involved in phosphotransferase system (PTS)**

| **Locus** | **Gene** | **Target carbohydrate** | **Log_2_ (Fold changes)** | | | **K number** |
| --- | --- | --- | --- | --- | --- | --- |
|  |  |  | **1 hpe** | **2 hpe** | **4 hpe** |  |
| SPSF3K_01731 | *ptsI* | Phosphoenol-pyruvate | - | -0.93 | -1.04 | K08483 |
| SPSF3K_00506 | *ptsG* | Glucose | -0.96 | -3.08 | -2.67 | K02779 |
| SPSF3K_00349 | *scrA* | Sucrose | - | 1.25 | 1.65 | K02810 |
| SPSF3K_01250 | *bglF* | b-glucosides | -0.82 | -1.41 | -1.49 | K02757 |
| SPSF3K_01353 |  |  | -2.68 | -1.04 | -0.71 |  |
| SPSF3K_01354 |  |  | -2.63 | -1.04 | - |  |
| SPSF3K_00116 | *treP* | Trehalose | - | - | -1.07 | K02819 |
| SPSF3K_01209 | *lacF* | Lactose | -0.81 | - | - | K02786 |
| SPSF3K_01210 | *lacE* |  | - | - | - | K02788 |
| SPSF3K_00352 | *celB* | Cellobiose | 1.35 | 1.01 | 0.78 | K02761 |
| SPSF3K_00826 |  |  | - | - | - |  |
| SPSF3K_01313 |  |  | -1.70 | - | -2.04 |  |
| SPSF3K_02084 |  |  | -1.71 | - | 1.61 |  |
| SPSF3K_02085 |  |  | -1.69 | - | - |  |
| SPSF3K_01469 | *cmtB* | Mannitol | -0.73 | - |  | K02798 |
| SPSF3K_01471 | *cmtA* |  | -1.14 | - |  | K02800 |
| SPSF3K_00942 | *manX* | Mannose | -2.05 | - | - | K02794 |
| SPSF3K_00943 | *manY* |  | - | - | - | K02795 |
| SPSF3K_00944 | *manZ* |  | -2.12 | - | - | K02796 |
| SPSF3K_00945 | *manXa* |  | - | - | - | K02793 |
| SPSF3K_02030 | *manX* |  | - | -1.68 | -1.79 | K02794 |
| SPSF3K_02031 | *manY* |  | - | - | - | K02795 |
| SPSF3K_02032 | *manZ* |  | - | - | - | K02796 |
| SPSF3K_00182 | *srlA* | Sorbitol | - | -4.11 | -3.46 | K02783 |
| SPSF3K_00183 | *srlE* |  | - | -4.09 | -3.61 | K02782 |
| SPSF3K_00184 | *srlB* |  | - | -3.42 | -3.28 | K02781 |
| SPSF3K_00119 | *sgaB* | ascrobate | - | -2.77 | -2.09 | K02822 |
| SPSF3K_00120 | *sgaT* |  | - | -2.89 | -2.35 | K03475 |
| SPSF3K_00157 | *sgaT* |  | - | -1.66 | - | K03475 |
| SPSF3K_00158 | *sgaB* |  | - | -1.50 | - | K02822 |
| SPSF3K_00159 | *sgaA* |  | - | - | 1.30 | K02821 |
| SPSF3K_01082 | *fruB* | Fructose | - | - | 1.15 | K02768 |
| SPSF3K_01200 | *fruA* |  | -0.81 | - | 0.65 | K02770 |

-, Not significant (|fold change| > 1.5 and FDR < 1e-5).
