# Supplementary material for: Transcriptome analysis unveils survival strategies of Streptococcus parauberis against fish serum
Source: PLoS One. 2021 May 26;16(5):e0252200. doi: 10.1371/journal.pone.0252200 (PMC8153452; doi:10.1371/journal.pone.0252200)
Supplement: S4 Table — (DOCX) [file pone.0252200.s005.docx]

**S4 Table. Gene expression of sortase A-mediated surface-anchored proteins containing LPXTG (or LPXTA) motif in C-terminal**

| **Gene name** | **Gene locus** | **Descriptions** | **Signal**  **peptide**  **motif** | **Log_2_ (Fold changes)** | | |
| --- | --- | --- | --- | --- | --- | --- |
|  |  |  |  | **1 hpe** | **2 hpe** | **4 hpe** |
| / | SPSF3K_00144 | Hypothetical protein | LPXTG | 1.63 | 2.22 | 2.24 |
| KEX1 | SPSF3K_00154 | Pheromone-processing carboxypeptidase | LPXTG | - | - | - |
| / | SPSF3K_00379 | C5a peptidase | LPXTG | 1.05 | 0.90 | 1.14 |
| / | SPSF3K_00426 | Antiphagocytic M protein | LPXTG | -1.00 | - | - |
| / | SPSF3K_00579 | Uncharacterized protein | LPXTG | 2.18 | - | - |
| / | SPSF3K_01115 | Hypothetical protein | LPXTG | 0.74 | - | - |
| DAN4 | SPSF3K_01126 | Cell wall protein | LPXTG | -1.18 | - | - |
| / | SPSF3K_01286 | Uric acid permease PucK | LPXTA | -1.32 | - | - |
| / | SPSF3K_01846 | Lactocepin | LPXTA | 1.68 | 0.97 | 1.47 |
| / | SPSF3K_02212 | Internalin-J | LPXTG | 0.78 | - | - |

-, Not significant (|fold change| > 1.5 and FDR < 1e-5).
